# miR-33a Expression Attenuates ABCA1-Dependent Cholesterol Efflux and Promotes Macrophage-Like Cell Transdifferentiation in Cultured Vascular Smooth Muscle Cells

**DOI:** 10.1155/2023/8241899

**Published:** 2023-06-16

**Authors:** Ikechukwu C. Esobi, Olanrewaju Oladosu, Jing Echesabal-Chen, Rhonda R. Powell, Terri Bruce, Alexis Stamatikos

**Affiliations:** ^1^Department of Food, Nutrition, and Packaging Sciences, Clemson University, Clemson, SC 29634, USA; ^2^Clemson Light Imaging Facility, Clemson University, Clemson, SC 29634, USA

## Abstract

Recent evidence suggests that the majority of cholesterol-laden cells found in atherosclerotic lesions are vascular smooth muscle cells (VSMC) that have transdifferentiated into macrophage-like cells (MLC). Furthermore, cholesterol-laden MLC of VSMC origin have demonstrated impaired ABCA1-dependent cholesterol efflux, but it is poorly understood why this occurs. A possible mechanism which may at least partially be attributed to cholesterol-laden MLC demonstrating attenuated ABCA1-dependent cholesterol efflux is a miR-33a expression, as a primary function of this microRNA is to silence ABCA1 expression, but this has yet to be rigorously investigated. Therefore, the VSMC line MOVAS cells were used to generate miR-33a knockout (KO) MOVAS cells, and we used KO and wild-type (WT) MOVAS cells to delineate any possible proatherogenic role of miR-33a expression in VSMC. When WT and KO MOVAS cells were cholesterol-loaded to convert into MLC, this resulted in the WT MOVAS cells to exhibit impaired ABCA1-dependent cholesterol efflux. In the cholesterol-loaded WT MOVAS MLC, we also observed a delayed restoration of the VSMC phenotype when these cells were exposed to the ABCA1 cholesterol acceptor, apoAI. These results imply that miR-33a expression in VSMC drives atherosclerosis by triggering MLC transdifferentiation via attenuated ABCA1-dependent cholesterol efflux.

## 1. Introduction

Atherosclerotic cardiovascular disease is currently the leading cause of mortality worldwide [1, 2]. The reason atherosclerotic cardiovascular disease leads to more deaths than any other disease globally is that atherosclerosis is known to be the predominant factor in death from myocardial infarctions and ischemic strokes [3]. Atherosclerosis is considered a chronic inflammatory condition which is the result of cholesterol accumulating within arterial wall cells and largely occurs in arteries located in bifurcated regions [4, 5]. Therefore, discovering mechanisms which contribute to cholesterol accumulation in arterial wall cells may lead to better therapies for atherosclerotic cardiovascular disease.

During the development of atherosclerosis, lipid primarily accumulates within intimal cells [6]. For decades, these lipid-laden intimal cells have been acknowledged as macrophages, mainly due to morphological and immunostaining analysis [7–9]. However, through cell lineage tracing technology and other cutting-edge methods, the majority of lipid-laden intimal cells found in atherosclerotic lesions are now recognized to be macrophage-like cells (MLC) of vascular smooth muscle cell (VSMC) origin [10–12]. Moreover, the VSMC-to-MLC phenotypic switch, coined transdifferentiation, can be triggered by VSMC cholesterol accumulation [13–15]. The transporter ABCA1 is mainly responsible for removing excess cholesterol from macrophages and other peripheral cells via apoAI-mediated cholesterol efflux [16]. ABCA1 is also required for regulating reverse cholesterol transport since functional ABCA1 is needed for nascent HDL synthesis [17]. Hence, ABCA1 expression is considered to be atheroprotective, and studies that have involved assessing atherosclerosis in the context of ABCA1 manipulation support this notion [18–20]. However, much of the published literature that has focused on analyzing the atherosclerotic impact of ABCA1 manipulation has been devoted to manipulating ABCA1 expression in macrophages and hepatocytes [21–23]. Interestingly, ABCA1 expression in MLC of VSMC origin has been shown to be reduced when compared to phenotypically normal VSMC and myeloid leukocyte-derived macrophages [12, 24, 25]. Mechanisms are poorly understood, though, why ABCA1 expression and subsequent apoAI-mediated cholesterol efflux do become impaired in VSMC that have transdifferentiated into a MLC phenotype [8]. The microRNA miR-33a is an intronic microRNA located within the transcription factor, sterol regulatory element-binding protein 2 (SREBP-2) [26, 27]. A major function of miR-33a is to silence expression of ABCA1, and this process is suggested to be proatherogenic by promoting cellular cholesterol retention within arteries [28]. However, many studies conducted that have assessed the therapeutic potential of inhibiting miR-33a have emphasized inhibiting only one of the mature strands of miR-33a (miR-33a-5p) [29]. Other studies involving miR-33a ablation in atherogenic mice have been contradictory [23, 30]. While some data suggests miR-33a expression is proatherogenic [31], miR-33a deletion has resulted in metabolic disturbances [32, 33], and these deleterious metabolic outcomes have also been shown by miR-33a-5p inhibition [34]. However, what does appear to be established is that ablating miR-33a in monocytes/macrophages exhibits atheroprotective effects via increasing ABCA1-dependent cholesterol efflux [35, 36]. However, the literature is scant on whether miR-33a expression in VSMC demonstrates proatherogenic properties and if any atherogenic effects from miR-33a are exacerbated in MLC of VSMC origin. The purpose of this work is to attempt to identify miR-33a atherogenic mechanisms in cultured VSMC. In this study, we aim to uncover any proatherogenic properties of miR-33a in phenotypically normal VSMC and VSMC that have converted into MLC through cholesterol loading. To rigorously assess the atherogenic potential of miR-33a expression in cultured VSMC/MLC, the immortalized mouse aortic smooth muscle cell line MOVAS cells [37, 38] were used to generate miR-33a knockout (KO) MOVAS cells. When compared to parental wild-type (WT) MOVAS cells which exhibit endogenous miR-33a expression, KO MOVAS cells were shown to demonstrate increased ABCA1 protein expression. Intriguingly, this increase in ABCA1 protein failed to enhance apoAI-mediated cholesterol efflux in KO MOVAS cells when retaining their VSMC phenotype. However, when we measured miR-33a-5p/3p expression in MOVAS WT cells in either nonhyperlipidemic conditions to maintain the VSMC phenotype or prohyperlipidemic conditions to induce the MLC phenotype, we observed a significant increase in miR-33a expression within the cultured MOVAS MLC versus phenotypically normal MOVAS cells. When we cholesterol-loaded MOVAS KO and WT cells to induce VSMC-to-MLC transdifferentiation, we detected increases in both ABCA1 protein and apoAI-mediated cholesterol efflux in the MOVAS KO cell groups. Lastly, when measuring expression of VSMC/macrophage markers in WT and KO MOVAS MLC exposed to apoAI, we observed delayed VSMC restoration in the WT MOVAS cell group. Thus, our results indicate that miR-33a expression in MLC impairs apoAI-mediated cholesterol efflux and implies that this atherogenic effect impedes restoration of these cells to their original VSMC phenotype.

## 2. Materials and Methods

### 2.1. Cell Culture Editing and Maintenance

MOVAS cells were obtained from the American Type Culture Collection (Manassas, VA, USA). These cells were used to generate KO MOVAS cells using the CRISPR-Cas9 system, with this gene editing work being completed as a fee-for-service by the Genome Engineering & Stem Cell Center (GESC@MGI) at Washington University in St. Louis. Briefly, two synthetic gRNA oligonucleotides with the following spacer sequences (5′-GCCGGCTGCACACCTCCTGG and 5′-CCTCTTTGGCCACGGCACCG) were purchased from the Integrated DNA Technologies (Coralville, IA, USA) and complexed with recombinant Cas9 protein acquired from the MacroLab at QB3-Berkeley before being nucleofected (Lonza, Basel, Switzerland) into MOVAS cells for deletion of the miR-33a gene. The transfected MOVAS cells were then single-cell sorted into 96-well plates, and single-cell clones were screened for miR-33a deletion by next-generation sequencing. KO MOVAS cells and parental WT MOVAS cells were cultured and maintained in standard growth medium consisting of high glucose Dulbecco's Modified Eagle's Medium (DMEM; Corning, New York, NY, USA), supplemented with FB Essence (10%; VWR Life Science Seradigm, Radnor, PA, USA), penicillin–streptomycin (P/S; 1%; Corning), and G418 (500 *μ*g/mL; VWR Life Science Seradigm). MOVAS cells were incubated at 37°C with 5% CO2 in 10 cm tissue culture dishes with standard growth medium changed every 2-3 days. For each respective experiment, MOVAS cells were first seeded into tissue culture plates and incubated in standard growth medium until reaching 70–80% confluency before exposing MOVAS cells to treatment medium, or continuing to culture cells in standard growth medium for cells in basal conditions.

### 2.2. Endpoint PCR and Endpoint RT-PCR

WT MOVAS cells and KO MOVAS cells were maintained under basal conditions in 6-well tissue culture plates (Corning) until reaching optimal confluency and then washed cells with phosphate-buffered saline (PBS; Corning) before extracting cellular gDNA using QuickExtract™ DNA Extraction Solution (Biosearch Technologies, Petaluma, CA, USA). Using cellular gDNA as a template, we performed endpoint PCR reactions (Q5® High-Fidelity DNA Polymerase; New England Biolabs, Ipswich, MA, USA) to assess deletion of the miR-33a gene in KO MOVAS cells by using a primer pair which flanked the miR-33a gene and analyzed the amplicons using a 2% TAE-agarose gel. To confirm miR-33a deletion in KO MOVAS cells, these PCR products were also purified and sequenced, with sequencing services performed by the Eton Bioscience, Inc. (San Diego, CA, USA). For endpoint RT-PCR, we allowed WT MOVAS cells and KO MOVAS cells to grow in 6-well tissue culture plates using a standard growth medium. Once cells reached optimal confluency, we washed cells with PBS (Corning), extracted total RNA using Direct-zol RNA purification kits (Zymo Research), and quantified the RNA using a SpectraMax® QuickDrop™ Micro-Volume Spectrophotometer (Molecular Devices, LLC., San Jose, CA, USA). We converted 100 ng of small RNA for each sample into cDNA by using a Quantabio qScript™ microRNA cDNA Synthesis kit (Beverly, MA, USA). The synthesized cDNA was used in carrying out endpoint RT-PCR with using a Quantabio PerfeCTa SYBR Green FastMix kit. The amplicons were evaluated using a 3.5% TBE-agarose gel. The primer pairs used for endpoint PCR and endpoint RT-PCR are listed in Table 1.

### 2.3. Cell Culture Treatments

We cultured MOVAS cells in serum-free medium (SFM) which consisted of high glucose DMEM, fatty acid-free bovine serum albumin (2 mg/mL; Sigma-Aldrich, Saint Louis, MO, USA), P/S, and G418. To induce hypolipidemic conditions, MOVAS cells were cultured in SFM for 72 hours before cell harvesting. To promote MOVAS cell transdifferentiation into a MLC phenotype, we also cultured cells in SFM with the addition of 10 *μ*g/mL cholesterol–methyl-*β*-cyclodextrin (M*β*CD:Chol) (Sigma-Aldrich, Saint Louis, MO, USA) for 72 hours [38]. To induce apoAI-mediated cholesterol efflux in MOVAS cells [38], we incubated MOVAS cells in SFM containing the apoAI (100 *μ*g/mL) cholesterol acceptor (Academy Bio-Medical Company, Houston, TX, USA) to use as an efflux medium.

### 2.4. RT-qPCR

We cultured and treated WT MOVAS cells and KO MOVAS cells in 6-well tissue culture plates. After treatments, we washed MOVAS cells with PBS (Corning) and then extracted and quantified total RNA as previously described for our endpoint RT-PCR reactions. For each sample, we converted 100 ng of total RNA into cDNA by using a Quantabio qScript® cDNA SuperMix kit for mRNA, and for small RNA, 100 ng of sample total RNA was converted into cDNA as described for endpoint RT-PCR. All synthesized cDNA was amplified using a Quantabio PerfeCTa SYBR Green FastMix kit. Our qPCR data was analyzed with the qTOWER^3^ G touch qPCR instrument (Analytik Jena US, Upland, CA, USA) and implementation of the *ΔΔ*CT method [39], with GAPDH being used as the housekeeping gene for mRNA and U6 used as the reference gene for small RNA. The primer pairs used for RT-qPCR are listed in Table 1.

### 2.5. Immunoblotting

Using PBS, we washed treated WT MOVAS cells and KO MOVAS cells cultured in 6-well tissue culture plates and collected lysates using RIPA lysis buffer containing mammalian protease inhibitors (VWR Life Science). We used a BCA assay (BioVision, Milpitas, CA, USA) for protein quantification, and equal amounts of protein among cell lysates were used for SDS-PAGE, and proteins were transferred onto PVDF membranes (Merck Millipore Ltd., Burlington, MA, USA). After blocking PVDF membranes, we probed for ABCA1 (1 : 1000 dilution, sc-58219; Santa Cruz Biotechnology, Dallas, TX, USA), SREBP-2 (1 : 500 dilution, sc-271616; Santa Cruz Biotechnology), GAPDH (1 : 1000 dilution, sc-365062; Santa Cruz Biotechnology), and HSP90 (1 : 5000 dilution, 610419; BD Biosciences, San Jose, CA, USA). After incubating the PVDF membranes in the primary antibodies, we used TBST for membrane washing and then incubated with horseradish peroxidase (HRP)-conjugated secondary antibody goat anti-mouse IgG (1 : 10,000 dilution, AP181P; Sigma-Aldrich), to detect ABCA1, SREBP-2, HSP90, and GAPDH. HRP was detected using ECL substrate reagent (Immobilon ECL Ultra Western HRP Substrate; MilliporeSigma, Billerica, MA, USA), and imaging analysis was performed with the aid of a ChemiDoc system (Analytik Jena US).

### 2.6. Cholesterol Efflux

KO MOVAS cells and WT MOVAS cells were seeded into 48-well tissue culture plates and incubated to optimal confluency within standard growth medium. To measure apoAI-mediated cholesterol efflux in KO MOVAS cells and WT MOVAS cells, we first washed cells with PBS and then incubated cells in SFM for 72 hours containing [^3^H] cholesterol (1 *μ*Ci/mL; PerkinElmer Life Sciences, Waltham, MA, USA). To induce MLC in a subset of MOVAS cells, we also incubated cells with 10 *μ*g/mL of M*β*CD:Chol (Sigma-Aldrich) during this 72 treatment duration [38]. After treatments, we removed cholesterol-loading medium, washed cells with PBS, and exposed cells to efflux medium for 72 hours to promote apoAI-mediated cholesterol efflux. After treatments, medium was filtered to remove nonadherent cells, and a liquid scintillation counter (LS 6500; Beckman Coulter, Brea, CA, USA) was used to count medium and cells. To calculate apoAI-mediated cholesterol efflux, we subtracted background cholesterol efflux in MOVAS cells incubated with medium not containing apoAI, as previously described [40, 41].

### 2.7. Cell Staining and Fluorescence Imaging

For all staining experiments, WT MOVAS cells and KO MOVAS cells were seeded into 4-chamber tissue culture slides (Corning) and grown to optimal confluency before being washed with PBS and beginning individual respective treatments which involved the following: (1) incubated in SFM with vehicle for 72 hours; (2) incubated in SFM with 10 *μ*g/mL of M*β*CD:Chol for 72 hours; (3) incubated in SFM with 10 *μ*g/mL of M*β*CD:Chol for 72 hours, washed with PBS, and exposed to efflux medium for 72 hours. For cellular stain preparations, MOVAS cells were prepared, stained, and imaged as described [38], with this work being conducted at the Clemson Light Imaging Facility (CLIF). Briefly, cells were incubated with ACTA2 (sc-32251; Santa Cruz Biotechnology) and CD68 (sc-20060; Santa Cruz Biotechnology) primary antibodies, then Alexa Fluor 546 goat anti-mouse IgG_2a_ (Invitrogen, Carlsbad, CA, USA) and Alexa Fluor 488 goat anti-mouse IgG_1_ (Invitrogen) secondary antibodies. Nuclear counterstaining was performed using DAPI (Invitrogen). Imaging was performed with a Leica SP8X MP Confocal System equipped with HyD detectors, a 405 nm laser, a tunable white light laser, and time gating capabilities (Leica Microsystems, Buffalo Grove, IL, USA). For imaging capture/export, a Leica LAS-X software (Leica Microsystems Version 3.5.5.19976) was used [38].

### 2.8. Statistical Analyses

We utilized SigmaPlot (Systat Software, Inc., San Jose, CA, USA) for statistical analysis. For all analyses, normality was determined using the Shapiro–Wilk test, and equal variance was assessed with the Brown–Forsythe test. For the analysis of two groups, a Student's *t*-test was used when both normality and equal variance were assumed, a Mann-Whitney *U* test was conducted when normality failed, and a Welch's *t*-test was performed when equal variance was violated. For >2 groups, we used a one-way ANOVA when both equal variance and normality were assumed and performed a Kruskal–Wallis one-way ANOVA on ranks when equal variance and/or normality were not met. A Tukey's test was used for parametric testing, and the Dunn's method was utilized for nonparametric testing. All studies requiring statistical analyses involved conducting three independent experiments and included either duplicate or triplicate biological replicates with data points shown and respective group means indicated by bars. Statistical significance is shown as a *P* value of <0.05.

## 3. Results

### 3.1. Characterizing KO MOVAS Cells

**Figure 1:**
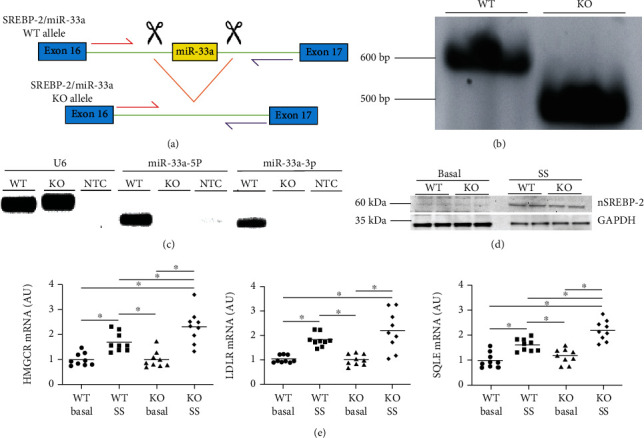
Characterization of KO MOVAS cells. Schematic illustrating miR-33a deletion in MOVAS cells (a) and endpoint PCR-based screening (b) demonstrating excision of miR-33a in KO MOVAS cells. The green line indicates intron, the red arrow indicates forward primer, and the purple arrow indicates reverse primer. (c) Endpoint RT-PCR for detection of the U6 reference gene, the miR-33a-5p mature strand, and the miR-33a-3p mature strand in WT and KO MOVAS cells. NTC = nontemplate control reactions. (d) Active nSREBP-2 protein and GAPDH housekeeping protein detected by immunoblotting in WT and KO MOVAS cells cultured in either basal or hypolipidemic serum-starved (SS) conditions. (e) SREBP-2 target gene expression measured by RT-qPCR in basal and SS WT and KO MOVAS cells. ^∗^ shows statistical significance (*P* < 0.05).

miR-33a is an intronic microRNA embedded in the SREBP-2 gene [26, 27]. To generate KO MOVAS cells, an approximate 100 bp partial intron sequence containing the miR-33a gene was excised from the SREBP-2 gene via CRISPR-Cas9 technology, and KO MOVAS cells were screened using endpoint PCR (Figures 1(a) and 1(b)). To further validate miR-33a being absent in KO MOVAS cells, we performed endpoint RT-PCR to assess expression of the miR-33a-5p and miR-33a-3p mature strands but failed to detect expression in KO MOVAS cells, which confirmed miR-33a deletion in this edited cell line (Figure 1(c)). Since SREBP-2 is essential for cellular cholesterol homeostasis [42], we evaluated SREBP.

Two functions in KO MOVAS cells to determine whether removing the intronic sequence containing miR-33a in KO MOVAS cells interfered with SREBP-2 functionality. When KO MOVAS cells were exposed to hypolipidemic conditions, we observed induction of active nSREBP-2 protein and increased SREBP-2 target gene expression that was similar to what was detected in WT MOVAS cells [38], also cultured in a hypolipidemic environment (Figures 1(d), 1(e)), which indicates KO MOVAS retain SREBP-2 function.

### 3.2. VSMC miR-33a Deletion Increases ABCA1 Protein Expression but Fails to Enhance apoAI-Mediated Cholesterol Efflux

**Figure 2:**
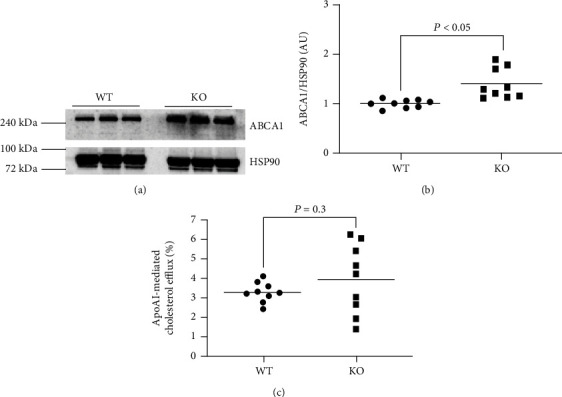
miR-33a expression in VSMC increases ABCA1 protein without enhancing apoAI-mediated cholesterol efflux. Representative immunoblot (a) for detection of ABCA1 protein and HSP90 housekeeping protein within lysates from WT and KO MOVAS cells cultured in basal conditions and immunoblotting quantification (b) of ABCA1 protein. (c) Cholesterol efflux measured in serum-starved, [^3^H] cholesterol-loaded WT and KO MOVAS cells incubated with apoAI.

miR-33a expression in several cell types has demonstrated the ability to impair apoAI-mediated cholesterol efflux via silencing ABCA1 protein expression [26, 28]. Moreover, we have shown that exosome-mediated transfer of anti-miR-33a-5p to VSMC also increases both ABCA1 protein and apoAI-mediated cholesterol efflux [43]. When we measured ABCA1 protein expression in WT MOVAS cells and KO MOVAS cells cultured in basal conditions, we detected increased expression of ABCA1 protein in the KO MOVAS cells (Figures 2(a) and 2(b)). Interestingly, when we measured apoAI-mediated cholesterol efflux in WT MOVAS cells and KO MOVAS cells with a normal (i.e., VSMC) phenotype, there was no change in apoAI-mediated cholesterol efflux between these two groups of MOVAS cells (Figure 2(c)).

### 3.3. Converting VSMC to a MLC Phenotype via Cholesterol-Loading Increases miR-33a Expression

**Figure 3:**
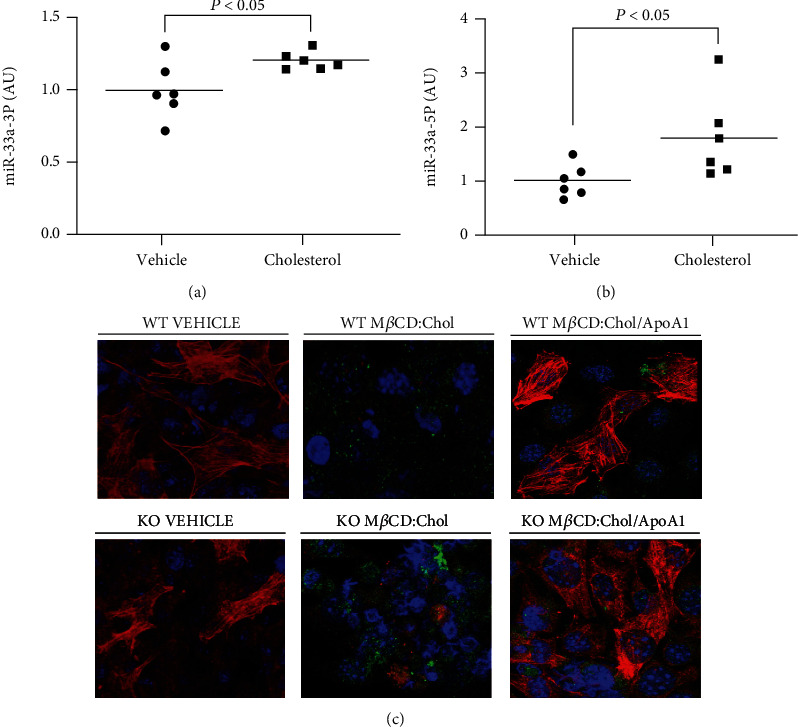
Converting MOVAS cells to a MLC phenotype increases miR-33a expression. MOVAS cells were cultured in serum-free medium containing either vehicle only to retain the VSMC phenotype or M*β*CD:Chol to induce VSMC-to-MLC transdifferentiation, and expression of miR-33a-3p (a) and miR-33a-5p (b) was measured in the treated cells via RT-qPCR. (c) WT and KO MOVAS cells were cultured in serum-free medium containing either vehicle only for VSMC maintenance or M*β*CD:Chol to promote MLC conversion. To revert MLC back to a VSMC phenotype, M*β*CD:Chol-loaded cells were subsequently exposed to apoAI. ACTA2 protein = red; CD68 protein = green; cell nuclei (DAPI) = blue.

It has been shown that VSMC which have transdifferentiated into a MLC phenotype exhibit reductions in ABCA1-dependent cholesterol efflux [12, 24, 25], but the mechanisms behind why this occurs are obscure [8]. To assess the impact of miR-33a expression on modulating VSMC-to-MLC transdifferentiation, we treated WT MOVAS with M*β*CD:Chol or vehicle only diluted in SFM. Intriguingly, we observed significant increases in miR-33a-5p and miR-33a-3p expressions in the cholesterol-loaded WT MOVAS MLC versus the vehicle-treated control WT MOVAS cells (Figures 3(a) and 3(b)). To attempt to elucidate any possible role of miR-33a in VSMC-to-MLC transdifferentiation, we first needed to assess the capacity of the gene-edited KO MOVAS cells to convert into MLC via cholesterol loading and then restoring VSMC phenotype through apoAI-mediated cholesterol efflux [38]. Similar to WT MOVAS cells, KO MOVAS cells were able to switch to a MLC phenotype upon M*β*CD:Chol exposure and revert back to a VSMC phenotype when incubated with apoAI (Figure 3(c)).

### 3.4. miR-33a Deficiency Increases Both ABCA1 Protein Expression and apoAI-Mediated Cholesterol Efflux in MLC

**Figure 4:**
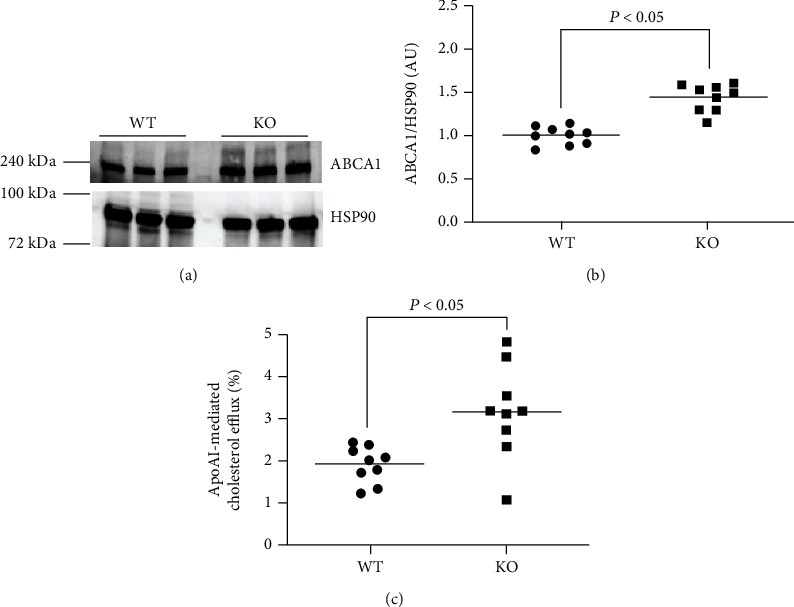
miR-33a expression in MLC impairs ABCA1-dependent cholesterol efflux. Representative immunoblot (a) for detecting ABCA1 protein and HSP90 housekeeping protein in lysates of WT and KO MOVAS MLC cultured in serum-free medium containing M*β*CD:Chol and immunoblot quantification (b) of ABCA1 protein. (c) Cholesterol efflux measured in serum-starved, M*β*CD:Chol-loaded, and [^3^H] cholesterol-loaded WT and KO MOVAS MLC exposed to apoAI.

miR-33a-deficient macrophages demonstrate enhanced apoAI-mediated cholesterol and increased ABCA1 protein expression [31, 35]. However, the effect of miR-33a expression on ABCA1-dependent cholesterol efflux in MLC of VSMC origin is unknown. To induce MLC transdifferentiation in MOVAS cells, we incubated WT MOVAS cells and KO MOVAS cells in M*β*CD:Chol, and measured ABCA1 protein expression in these cells. We detected a significant increase in ABCA1 protein within the KO MOVAS MLC when compared to the WT MOVAS MLC (Figures 4(a) and 4(b)). To test whether this increase in ABCA1 protein expression enhanced apoAI-mediated cholesterol efflux in KO MOVAS MLC, we also exposed WT MOVAS MLC and KO MOVAS MLC to apoAI. In the KO MOVAS MLC, we observed enhanced apoAI-mediated cholesterol efflux in these cells (Figure 4(c)), which implies an increase in ABCA1-dependent cholesterol efflux within the KO MOVAS MLC.

### 3.5. miR-33a Expression Delays VSMC Restoration in Cholesterol-Loaded MOVAS Cells Exposed to apoAI

**Figure 5:**
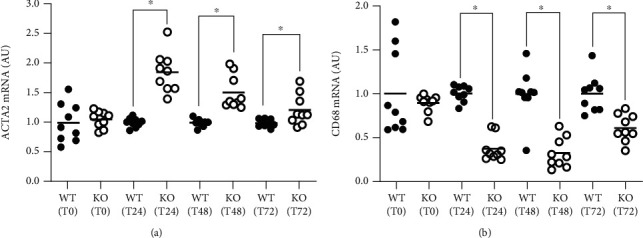
miR-33a ablation in MLC of VSMC origin rapidly restores VSMC in the presence of apoAI. To trigger VSMC-to-MLC transdifferentiation, WT, and KO MOVAS cells were cultured in cultured in serum-free medium containing M*β*CD:Chol. MOVAS cells were subsequently exposed to apoAI to induce cholesterol efflux for 24, 48, or 72 hours, and ACTA2 (a) and CD68 (b) expressions were measured within the apoAI-treated and baseline (T0) cells by using RT-qPCR. ^∗^ shows statistical significance (*P* < 0.05).

Intimal VSMC-to-MLC transdifferentiation is thought to contribute to atherosclerosis progression, and this process can be triggered by VSMC cholesterol accumulation [13]. Thus, the removal of excess intracellular cholesterol from MLC that is of VSMC origin may be atheroprotective via restoring the VSMC phenotype. We converted WT MOVAS cells and KO MOVAS cells to MLC by cholesterol-loading cells using M*β*CD:Chol and then exposed MOVAS MLC to apoAI to promote cholesterol removal via inducing apoAI-mediated cholesterol efflux. When we measured mRNA expression of the classical VSMC marker ACTA2 (Figure 5(a)) and classical macrophage marker CD68 (Figure 5(b)) in MOVAS MLC incubated with apoAI, we reported increased ACTA2 expression and decreased CD68 expression in the KO MOVAS MLC versus the WT MOVAS MLC. Hence, these findings indicate that cholesterol-loaded KO MOVAS cells can rapidly restore their VSMC phenotype in the presence of apoAI.

## 4. Discussion

In this study, we wanted to determine the role of VSMC miR-33a expression on ABCA1-dependent cholesterol efflux and VSMC-to-MLC transdifferentiation. To directly test this, we needed to utilize cultured VSMC exhibiting endogenous miR-33a expression (WT MOVAS cells) [38] and miR-33a-deficient cultured VSMC (KO MOVAS cells). While we detected increased ABCA1 protein expression in KO MOVAS cells, this increase in ABCA1 protein failed to significantly enhance apoAI-mediated cholesterol efflux. However, in KO MOVAS MLC, we did observe increases in both ABCA1 protein expression and apoAI-mediated cholesterol efflux when compared to WT MOVAS MLC. This increase in ABCA1-dependent cholesterol efflux observed in MLC and not VSMC may have been at least partially due to increased miR-33a-5p/3p expression detected in the WT MOVAS MLC compared to WT MOVAS cells, since higher miR-33a expression would have a more prominent effect on silencing ABCA1. Another important finding we reported was WT MOVAS MLC demonstrating delayed VSMC restoration when exposed to apoAI, which suggests that miR-33a expression in cholesterol-filled VSMC prevents restoring the VSMC phenotype by impairing ABCA1-dependent cholesterol efflux.

Assessing the proatherogenic impact of miR-33a has previously been rigorously investigated [21, 22]. However, this data is inconclusive as to whether systemic miR-33a inhibition is truly atheroprotective [34, 36, 44]. Instead, miR-33a ablation in certain cell types appears to have a stronger antiatherogenic effect than global miR-33a inhibition and arguably the most prominent atheroprotective effect occurs in macrophages [23]. However, while lipid-laden intimal macrophages have traditionally been recognized to be the predominant cell type contributing to atherosclerosis, there has been a recent shift in this paradigm, as it is now also acknowledged that VSMC/MLC play a large role in the development of atherosclerosis, too [12, 45]. And while we previously have shown that anti-miR-33a-5p delivery to cultured VSMC increased ABCA1 protein and apoAI-mediated cholesterol efflux [43], there has been no prior published literature (to our knowledge) that has rigorously tested the effect of miR-33a expression in MLC of VSMC origin on ABCA1-dependent cholesterol efflux. Our results did show that miR-33a expression did decrease ABCA1-dependent cholesterol efflux in cultured MLC, which does provide a possible mechanism why ABCA1 expression and apoAI-mediated cholesterol efflux are shown to be impaired in MLC originating from VSMC and portrays a potential proatherogenic effect of VSMC/MLC miR-33a expression. We were intrigued that converting WT MOVAS cells to a MLC phenotype via M*β*CD:Chol loading resulted in increased miR-33a-5p/3p expression, since cholesterol accumulation in various cell types has been shown to decrease miR-33a expression because miR-33a is coexpressed with its host gene SREBP-2, which regulates cholesterol biosynthesis [46–49]. We recognize that this effect may be specific for MOVAS cells being an immortalized VSMC line and so may react to excess cholesterol accumulation differently than other types of primary VSMC. We also acknowledge that while expression of miR-33a in WT MOVAS cells was shown to be increased upon M*β*CD:Chol exposure, miR-33a expression levels may eventually decline in MOVAS MLC if these cells continue to be exposed to prolonged hypercholesterolemic conditions. Another possibility is that the VSMC-to-MLC phenotypic switching which occurs in M*β*CD:Chol loading WT MOVAS cells may robustly stimulate miR-33a expression to levels that are similar to macrophages, which are cells that have higher miR-33a expression as opposed to other vascular cells (e.g., endothelial cells) [28]. Hence, this type of miR-33a induction in MOVAS MLC may potentially augment miR-33a expression to greater levels than that are normally detected in WT MOVAS cells. Moreover, since MLC resembles and may behave similarly to monocyte-derived macrophages under certain conditions, it would be intriguing to decipher whether MLC of VSMC origin are also capable of exhibiting M1 and M2 macrophage qualities, since this would likely impact atherosclerosis development [50–52]. Thus, future studies should assess if this phenomenon of MLC demonstrating M1 and/or M2 properties occurs and if this is mediated by miR-33a expression. If future findings do demonstrate that miR-33a expression in MLC of VSMC origin is able to alter polarization in these cells, then this may change the cell phenotype that results in these cells being proatherogenic via negatively impacting autophagy [53] as well. Indeed, assessing these endpoints should also be addressed in future experiments.

In our study, there are some limitations we want to address. One limitation is not attempting to identify why we did not observe an increase in apoAI-mediated cholesterol efflux in KO MOVAS cells despite a significant increase in ABCA1 protein detected in these cells. This was an interesting finding, as we have previously reported that exosome-mediated delivery of anti-miR-33a-5p to primary VSMC increases ABCA1-dependent cholesterol efflux [43]. Future studies should be conducted in KO MOVAS cells and WT MOVAS cells to distinguish between total ABCA1 protein and active cell-surface ABCA1 [54] to determine whether active ABCA1 protein levels are similar in these cells, which would explain why there was no change in apoAI-mediated cholesterol efflux in these two groups. Another limitation for our study is not further delineating the effect of miR-33a on VSMC-to-MLC transdifferentiation by performing transfection studies in the KO MOVAS MLC to determine whether VSMC restoration is delayed in these cells when being introduced to miR-33a. Therefore, future experiments should be performed which involve transfecting miR-33a-deficient VSMC/MLC with miR-33a-5p and/or miR-33a-3p to assess the potential impact these two mature strands have on possibly altering VSMC-to-MLC transdifferentiation. We also acknowledge that a limitation to our study is only attempting to identify miR-33a-mediated suppression of ABCA1 in triggering VSMC-to-MLC phenotypic switching within a hyperlipidemic environment. Since VSMC-to-MLC transdifferentiation appears to be a very complex process [55] involving other microRNAs [15] besides miR-33a as well as NOTCH signaling [56], it is quite likely that the mechanisms which modulate MLC transdifferentiation are convoluted and possibly intricately related. Hence, future studies which manipulate other biological processes thought to be involved in VSMC-to-MLC transdifferentiation along with simultaneously altering miR-33a in VSMC/MLC should be conducted. Lastly, our experiments should be repeated using Cre-Lox technology to effectively delete miR-33a in cultured primary VSMC [57–59], as these studies may provide more biologically relevant findings when compared to data generated from MOVAS cells, which are an immortalized VSMC line [60].

## 5. Conclusions

**Figure 6:**
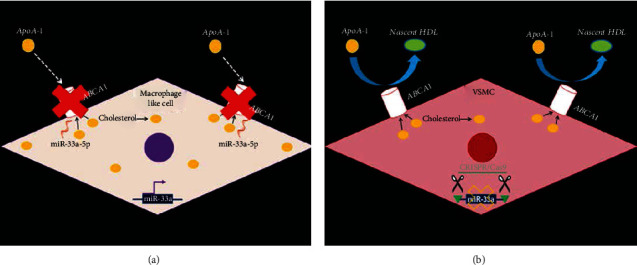
Proatherogenic model of VSMC miR-33a expression: (a) Expression of miR-33a-5p/3p in VSMC silences ABCA1 which allows cholesterol levels to accumulate in VSMC and prevents excess intracellular cholesterol to be removed via apoAI-mediated cholesterol efflux, triggering VSMC-to-MLC transdifferentiation. (b) MiR-33a ablation in VSMC hinders miR-33a-mediated suppression of ABCA1, allowing robust ABCA1-dependent cholesterol efflux to remove high levels of cellular cholesterol, thereby protecting VSMC from MLC phenotype switching and promoting VSMC restoration upon apoAI exposure.

Our results illustrate a proatherogenic mechanism for VSMC/MLC miR-33a expression (Figure 6). Indeed, our findings depict miR-33a expression as being atherogenic in cultured MLC of VSMC origin via impairing ABCA1-dependent cholesterol efflux in these cells. Furthermore, in cultured VSMC that have transdifferentiated into MLC from excess cholesterol accumulation, miR-33a expression delays VSMC restoration in these cholesterol-laden cells when abundant apoAI is available. Therefore, these results represent intimal VSMC/MLC miR-33a expression as an atherogenic driver, and so a possible atheroprotective intervention may be miR-33a inhibition specifically within intimal VSMC/MLC. However, future studies assessing atherosclerosis in WT mice and VSMC-specific miR-33a KO mice on an atherogenic background should be conducted first to deduce whether VSMC miR-33a expression is proatherogenic in vivo.

## Figures and Tables

**Table 1:** Primer pairs.

Target		Sequence (5′–3′)
GAPDH	Forward: Reverse:	CGTGCCGCCTGGAGAAAC TGGGAGTTGCTGTTGAAGTCG
ACTA2	Forward: Reverse:	GCTTCGCTGGTGATGATGCTC AGTTGGTGATGATGCCGTGTTC
CD68	Forward: Reverse:	CTTCCCACAGGCAGCACA ATGATGAGAGGCAGCAAGAGG
miR-33a flank site	Forward: Reverse:	CTTCCCACAGGCAGCACA ATGATGAGAGGCAGCAAGAGG
Mouse U6 snRNA	Forward:	TGGCCCCTGCGCAAGGATG
miR-33a-5p	Forward:	CAATGTTTCCACAGTGCATCA
miR-33a-3p	Forward:	CAATGTTTCCACAGTGCATCA
Global small RNA	Reverse:	GCATAGACCTGAATGGCGGTA
HMGCR	Forward: Reverse:	AGGCAGACTGCAAGGACAAG CCGTGAATGCAGGAGCCATC
SQLE	Forward: Reverse:	GCTGGGCCTTGGAGATACAG CAGTGGGTACGGAATTTGAACT

## Data Availability

All data represented in this study is contained within the manuscript.
